# Electrodeposition of 4-Benzenesulfonic Acid onto a Graphite-Epoxy Composite Electrode for the Enhanced Voltammetric Determination of Caffeine in Beverages

**DOI:** 10.1155/2019/8596484

**Published:** 2019-01-23

**Authors:** Leonardo de A. Furtado, Mariana C. de O. Gonçalves, Carlos V. M. Inocêncio, Edilson M. Pinto, Daniela de L. Martins, Felipe S. Semaan

**Affiliations:** ^1^Laboratório Aniy K. Ohara de Sensores Compósitos e Eletroanálise, Departamento de Química Analítica, Universidade Federal Fluminense, Campus do Valonguinho, Prédio do Instituto de Química, Centro, Niterói, RJ 24020-141, Brazil; ^2^FAIP, Marília, São Paulo, Brazil; ^3^Grupo de Pesquisas em Catálise e Síntese (Laboratório 413), Departamento de Química Orgânica, Universidade Federal Fluminense, Campus do Valonguinho, Prédio do Instituto de Química, Centro, Niterói, RJ 24020-141, Brazil

## Abstract

Caffeine is widely present in food and drinks, such as teas and coffees, being also part of some currently commercialized medicines, but despite its enhancement on several functions of human body, its exceeding use can promote many health problems. In order to develop new fast approaches for the caffeine sensing, graphite-epoxy composite electrodes (GECE) were used as substrate, being modified by different diazonium salts, synthetized as their tetraflouroborate salts. An analytical method for caffeine quantification was developed, using sware wave voltammetry (SWV) in Britton–Robinson buffer pH 2.0. Detection limits for bare electrode and 4-benzenesulfonic modified electrode were observed circa 145 *µ*mol·L^−1^ and 1.3 *µ*mol·L^−1^, respectively. The results have shown that the modification shifts the oxidation peaks to lower potential. Kinetics of the reaction limited by diffusion was more expressive when caffeine was added to the solution, resulting in decreases of impedance, characterized by lower *R*_ct_. All results for caffeine determination were compared to a reference chromatographic procedure (HPLC), showing no statistical difference. Analytical parameters for validation were suitably determined according to local legislation, leading to a linear behaviour from 5 to 150 *µ*mol·L^−1^; precision of 4.09% was evaluated based on the RDC 166/17, and accuracy was evaluated in comparison with the reference method, with recovery of 98.37 ± 2.58%.

## 1. Introduction

Caffeine (3,7-dihydro-1,3,7-trimethyl-1H-purine-2,6-dione), also known as 1,3,7-trimethylxanthine, is an alkaloid from the methylxanthines group [1]. It can be found in nature, in many sorts of plants such as mate and tea leaves and coffee beans [2].

Food and pharmaceutical industries use caffeine in wide products, ranging to teas, coffees, energy and soft drinks, as well as, in medications [3]. Its consumption provides enhance on the performance of human body since it acts as stimulant for the cardiac muscle, gastric secretion, diuresis, central nervous, and respiratory systems [3–5]. Due to all those advantages, caffeine is consumed in higher quantities for athletes to enhance their physical performance and mental aptitude; however, the exceeding use of caffeine is considered doping and it is forbidden by the World Anti-Doping Agency (WADA) [6].

Caffeine is generally safe when consumed in low to moderate quantities. Nevertheless, considering its action as a central nervous system stimulant (as a result of its operation over a variety of molecular targets such as adenosine receptor, calcium channels, GABA_A_ receptors, and phosphodiesterases), its excessive consumption can culminate in undesirable effects. Significant increase in the risk of low birth weight is associated with a high caffeine intake during pregnancy. Additionally, dependence, tolerance, tremor, anxiety, insomnia, weakness, palpitations, gastrointestinal disturbances, and increased blood pressure are some of the disorders caused by ingesting caffeine in large amounts [6, 7]. The consumption of caffeine above 200 mg/day may lead to death, since this quantity is already considered toxic [7]. Therefore, the development of a high-quality method to quantify caffeine in different samples is of paramount importance. Many techniques have been explored over the years; for instance, ultraviolet-visible (UV-Vis) spectrophotometry [8], high-performance liquid chromatography (HPLC) [9–11], capillary electrophoresis (CE) [12, 13], FT-Raman spectrometry [14], near-infrared spectroscopy (NIRS) [15], and gas chromatography (GC) [16]. In comparison to all these techniques cited, voltammetry presents good features that encourage the increase in its use. The technique accounts with a satisfactory sensitivity, lower costs, lower time-consuming, ease of operation, and regeneration, besides the fact that it can be also applied to coloured and hypersaline samples [17–21].

Devaramani's group has developed an electrochemical sensor for dopamine based on a graphite pencil lead electrode (GPLE) modified with a covalently anchored multilayer of 4-aminobenzene sulfonic acid [22]. Tefera and co-workers applied square-wave voltammetry to the simultaneous determination of paracetamol and caffeine employing a poly(4-amino-3-hydroxynaphthalene sulfonic acid)-modified glassy carbon electrode [23]. Both the Tefera and Devaramini groups employed sulfonic acids as electrode modifiers in the detection of organic bases. Gupta's group also developed a modified sensor aiming food analysis [24]. Encouraged by these outcomes, we envisaged to construct an *ex situ* chemically modified electrochemical sensor for caffeine based on the modification of a composite graphite-epoxy electrode covalently bound to aromatic organic acids. Linkage between the organic modifier and the electrode surface was achieved using the electrochemical reduction of substituted tetrafluoroborate arenediazonium salt as platform [25]. Lo and co-workers have applied diazonium salts as a versatile molecular glue, being an intermediate to achieve more complex sensors modifications [26]. Diazonium salt modification was also used, in a previous work, to create a 4-benzenesulfonic acid modified graphite-epoxy composite electrode, which has enhanced the sensibility and detection limits for lead, cadmium, and zinc [27].

This paper presents a new method for the determination of caffeine through voltammetry using the square wave technique. It presents the modification procedure, the study of pH used for buffer solution's choice, and an electrochemical impedance spectroscopy (EIS) study of all modifications in presence and absence of the analyte, as well, analytical comparison with bare and modified electrodes for caffeine determination. To validate the method, real samples of drinks sold in Brazil (green tea, black tea, and guarana beverage with ginseng aroma) were assessed by the proposed procedures and compared to an HPLC method. Further parameters such as accuracy, precision, linear range-linearity, and detection limit were calculated based on a local guide from the National Agency of Sanitary Vigilance (ANVISA) [28]. AFM images were measured to evaluate the surface topography and roughness, before and after the modification.

## 2. Materials and Methods

### 2.1. Chemicals

Unless specified, all the reagents were of analytical grade. Unless otherwise mentioned, all the reagents were employed as aqueous solutions (bi-distilled water), used without further purification and purchased from VETEC (Brazil). Glacial acetic acid was purchased from Merck (Germany). NaOH aqueous solutions (6.0 mol·L^−1^) were employed to adjust the pH values (NaOH from Merck, Germany). Britton–Robinson buffer (BRB) (0.04 mol·L^−1^) was used as support electrolyte and prepared by mixing equal parts of 0.04 mol·L^−1^ solutions of acetic acid, boric acid, and phosphoric acid. Graphite powder (<20 *µ*m, Sigma-Aldrich, United States of America) and epoxy resin (Avipol, Brazil) were utilized for preparing the working electrodes. Diazonium salt solutions were prepared by dissolving the salts in an HCl solution (from Merck, Germany, 0.1 mol·L^−1^).

### 2.2. Preparation of the Composite Electrodes

The graphite-epoxy composite electrodes were prepared as reported in the literature [29, 30]: epoxy resin (35%) and graphite (65%) were mixed, in mass, until obtaining a homogeneous mixture. This mixture was placed into a small syringe modified with a copper wire for the electrical contact, covered up by the piston and put under pressure (24 h) to cure the polymer. After the curing time, the composite electrode was sanded with crescent mesh sandpaper (from 600 to 1500 mesh) for polishing of the surface.

### 2.3. Preparation of the Diazonium Salts

In a beaker equipped with a magnetic stirring bar, the aromatic amine (0.01 mol) was dissolved in a solution of hydrochloric acid (2.5 mL HCl 37%, 2.5 mL water). The resulting mixture was cooled to –10°C with the aid of a cooling bath (ice/water, CaCl_2_). An aqueous solution of sodium nitrite (0.7 g on 1.5 mL water) was dropped onto the amine solution under vigorous stirring, while keeping the reaction temperature below–5°C. After the addition, the mixture was further stirred for 20 min (*T* < −5°C). Subsequently, an aqueous solution of sodium tetrafluoroborate (1.5 g on 3.0 mL water) was dripped onto the reaction mixture under temperature control (*T* < −5°C). After completed addition, the reaction mixture was stirred for an additional 30 min. The solid obtained was filtered off and washed with cold water. After drying, the product was characterized by IR spectroscopy.

### 2.4. Instrumental and Software

All voltammetric analyses were performed using Ivium® CompactStat potentiostat (Ivium, the Netherlands) and Iviumsoft® software, version 2.699. In order to assemble the electrochemical cell, a labmade Ag|AgCl (3 mol·L^−1^ KCl) was used as reference electrode, a platinum wire (LabSolution®, Brazil) was used as counter electrode, and the graphite/epoxy composite electrode, bare or modified, was used as working electrode.

HPLC measurements were executed on a Shimadzu® equipment (Prominence®, software LC solutions 1.0.0.1, Japan). A C18 column (Shim-pack VP-ODS, 250 × 4.6 mm, Japan) was employed with methanol/water (1 : 1) (0.600 mL/min) as the mobile phase.

All the atomic force microscopy (AFM) measurements were performed with a FlexAFM instruments (Nanosurf®, Switzerland) and controlled by Nanosurf Easyscan 2 software. The probe used was EZ2-Flex AFM (100 *µ*m) and cantilever model PPP-nclr. The figures were assembled using Gwyddion® software (Czech Republic).

### 2.5. Sample Preparation

Four samples were chosen in order to apply the method developed in the present work to commercial sources of caffeine: three samples of teas (tea bags: two samples of black teas and one of green tea) and one sample of guarana drink with ginseng aroma. The guarana drink was used without any kind of chemical treatment before the voltammetric analyses. For the HPLC, samples of guarana drink were filtered with a membrane (20 *µ*m) and diluted by half. The teas were made in 50 mL beakers with distilled water at approximately 90°C. The sachets were infused for 3 to 5 minutes. This solution was adjusted to 50.0 mL and used directly for voltammetric determination. For HPLC, the samples were diluted from 20 to 50 times after being filtered in a membrane (20 *µ*m).

### 2.6. Electroanalytical Procedures

#### 2.6.1. Electroactive Surface of Work Electrodes

To test the performance of the composite electrodes, cyclic voltammetry was accomplished by varying the scan rates (from 100 to 10 mV/s), with 10 mV of potential step. As the electrolyte, a solution of KCl 0.5 mol·L^−1^ was used and 5 mmol·L^−1^ of ferricyanide was added to evaluate the electroanalytical performance of the composite electrode.

#### 2.6.2. Cyclic Voltammetry for the Deposition of the Aromatic Organic Molecules

Solutions of the diazonium salts (5 mmol·L^−1^) were used for modifying the electrode surfaces. Parameters for salt deposition: 15 cycles; scan rate 50 mV·s^−1^; 10 mV of potential step; from 0.4 V to −1.0 V.

#### 2.6.3. Cyclic Voltammetry for Evaluation of Modified Electrodes on Caffeine Determination

For evaluation of the modified electrodes, cyclic voltammetry was realized in a 25.0 mL cell containing 0.04 mol·L^−1^ BRB with pH values of 2, 4, 6, 8, and 10. The parameters were as follows: 1.0 to 2.0 V potential window; 100 mV·s^−1^ of scan rate; 10 mV of potential step. Measures were made in blank BRB solution and BRB solution containing 2 mmol·L^−1^ of caffeine.

#### 2.6.4. Square Wave Voltammetry Procedure for Caffeine Determination

The cell contained 20.0 mL of 0.04 mol·L^−1^ BRB (pH = 2). For the determination of caffeine, an analytical curve was generated as follows: additions of 0.05 mL and 0.10 mL of caffeine standard 5 × 10^−3^ mol·L^−1^. The optimization of the parameters for the determination of caffeine will be explained Results and Discussion. The parameters chosen were as follows: potential window: 0.8 to 2.0 V; pulse amplitude: 100 mV; frequency: 12 Hz; potential step: 10 mV. To determinate the caffeine content in the studied samples, 0.50 mL of the prepared samples were added to 20.0 mL of 0.04 mol·L^−1^ BRB, followed by 4 additions of 0.10 mL of caffeine standard 5 × 10^−3^ mol·L^−1^. The caffeine contents in samples were calculated by standard addition curves. All samples were measured in triplicate.

### 2.7. HPLC Procedures

The HPLC procedures were made based on an application note from Agilent Technologies [31], with slight adjustments. An analytical curve of caffeine was constructed with four points of concentration: 5, 10, 15, and 20 ppm. Caffeine peak was recorded around 7.5 minutes of sweep, both in standard as in samples. For the sample readings, each run had a total time of 15 minutes to ensure full sample passage by the column.

## 3. Results and Discussion

### 3.1. Composite Electrode Manufacture and Evaluation

The composite electrodes were fabricated as mentioned in the experimental section. The evaluation of the electrodes was based on previous works [29, 30], via cyclic voltammetry. The electrodes were tested against an [Fe(CN)_6_]^−3^/[Fe(CN)_6_]^−4^] system, with potassium chloride as the electrolyte. Based on the results, the electrode has shown reproductive responses and electroanalytical areas ((0.139 ± 0.006) cm^2^; *n* = 20), even after several renovations of the surface. They also displayed a linear dependence between cathodic and anodic currents and the scan rates square, according to Randles–Sevcik equations (*i*_p_ = 0.306 − 0.039; *r*^2^ = 0.991 and *i*_p_ = − 0.315 + 0.006; *r*^2^ = 0.992). The composite electrodes showed to be of great analytical capability, especially when considering the potential for modifications, besides the low costs and stability.

### 3.2. Diazonium Salts Synthesis

**Scheme 1:**
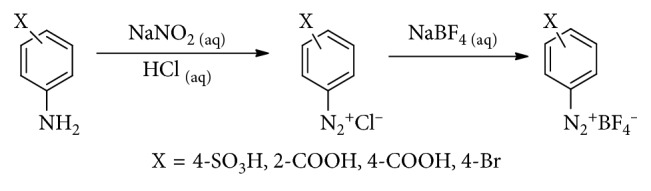
Preparation of tetrafluoroborate arenediazonium salts.

Four different diazonium compounds were synthesized using a methodology previously described [32, 33], but instead of direct use for modification as proposed in these papers, they were synthesized as their tetrafluoroborate salts [34]. The synthesis reaction is described in scheme 1. Arenediazonium salts were characterized via IR spectroscopy. All diazonium salts presented bands of medium intensity between 2300 and 2200 cm^−1^ which were assigned to the stretching mode ν (N≡N). Bands of wavelengths between 3500 and 3300 cm^−1^ were absent from the diazonium salts spectrum, indicating the products were free from aromatic amine contamination as these bands are characteristics of the stretching mode ν (N-H).

### 3.3. Electrode Modification with Diazonium Salts

Modifications of the electrodes by arenediazonium salts were carried out based on the methodology described in the literature [35] and adapted to the graphite-epoxy electrode [32, 36]. The main difference between the methodology found in the literature and the one optimized is the number of cycles (15) and the absence of organic solvents, since only a 0.1 mol·L^−1^ HCl solution was utilized to dilute the salts.

**Scheme 2:**
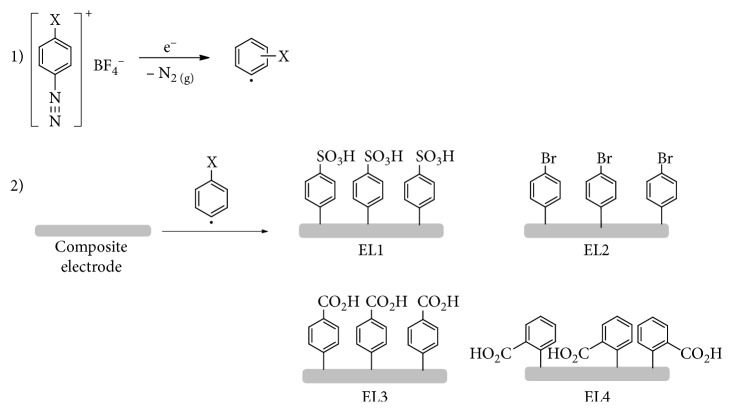
Reduction processes for the diazonium salts, followed by the reaction with the electrode surface.

For chemical modification, cyclic voltammetry was carried out from 0.4 to −1.0 V, being observed a reduction peak (attributed to the diazonium salt) around −0.6 V, varying slightly (±0.1 V) according to the used salt. In Scheme 2, the proposed reductions for the assessed salts are shown. The resulting radical, from the reduction process, would react covalently with the electrode surface. Each different modification received a name from EL1 to EL4 to easy identification.

**Figure 1:**
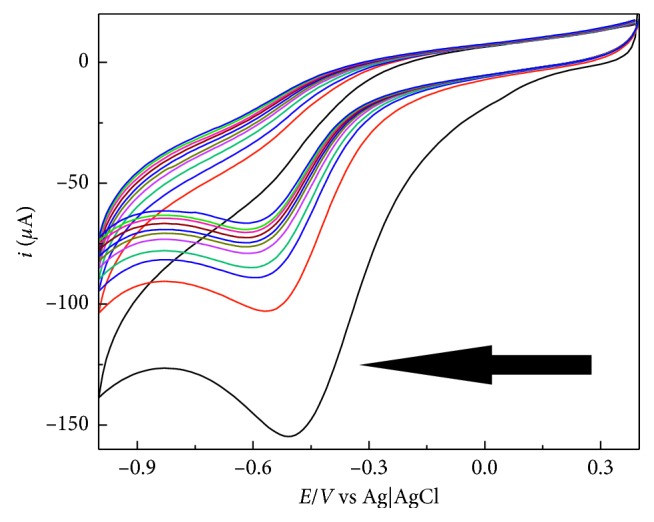
Typical result from the modification process (from the first to the following voltammetric cycles, until the obtainment of surface modification) with diazonium salts, in particular, the result to EL1 modification is shown. The arrow shows the scan direction and the first cycle of the process.

An example of the voltammograms obtained during modification is shown in Figure 1. As it can be observed, the modification process would take around 6 to 9 cycles to finish. This is the main reason that a higher number of cycles is made. It is also important to point out that the same 5 mmol·L^−1^ diazonium salt solution can be used multiple times, for a couple of days, being considered as stable. After several days, diazonium salts start to spontaneously decompose and a new solution must be prepared. All four synthesized diazonium salts were used to modify the composite electrodes, and their applicabilities to determine caffeine were then evaluated.

### 3.4. Evaluation of Modified Electrodes for Caffeine Determination

**Figure 2:**
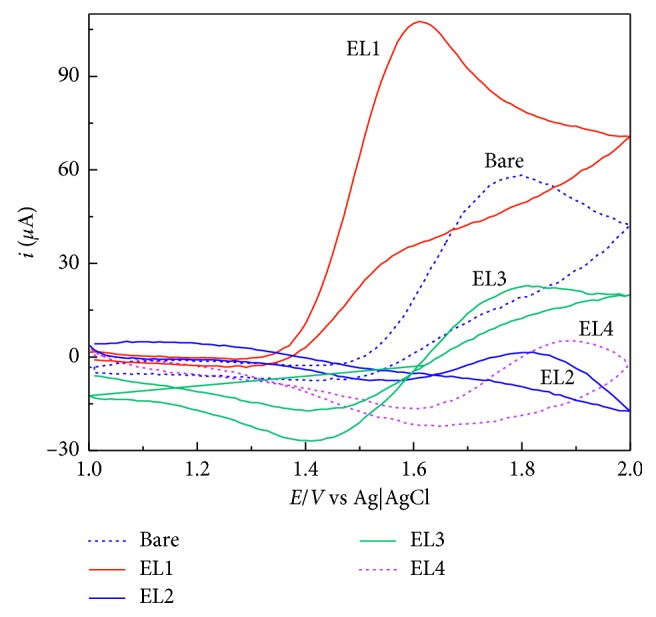
Cyclic voltammograms obtained for each modified electrode in presence of 2 mmol·L^−1^ of caffeine, in 0.04 mol·L^−1^ BRB pH 2.0. Cyclic voltammetry parameters: scan rate 100 mV·s^−1^; potential step of 10 mV. The arrow shows the scan direction.

Cyclic voltammetry was accomplished in BRB solution with pH values from 2.0 to 10.0, to determinate if and how each modification would benefit caffeine assessment, in comparison to the bare composite electrode. The results have shown that the best overall system was the modification with 4-sulfobenzenediazonium salt (EL1), the most acidic modifier, in pH value of 2.0.  Figure 2 shows cyclic voltammograms for caffeine onto different modified electrodes and a bare one, all in pH value of 2.0.

Based on the results, the electrode modified with 4-sulfobenzenediazonium salt not only exhibited an increase in the sensibility but also a shift in the oxidation potential from 1.80 to 1.60 V. The other modified electrodes have also displayed a less relevant shift in the oxidation potential, with a sensitivity loss, when comparing to the bare electrode.

From these results, two paths were chosen to further study the system. The first was to explore all conditions by electrochemical impedance spectroscopy in pH 2.0, using the open circuit potentials (OCP) and the oxidation potential of each one, with and without caffeine. The second path was to evaluate the analytical application of the bare electrode and electrode modified by 4-sulfobenzenediazonium for caffeine determination.

### 3.5. Assessment of Interfacial Behaviour

**Figure 3:**
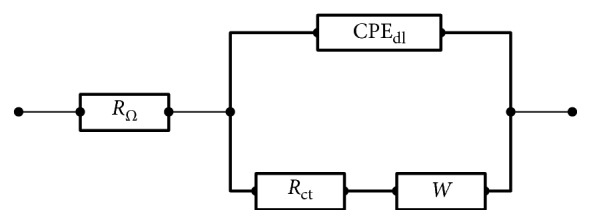
Modified Randles circuit used to fit the spectra in complex plane, which presents a cell resistance (*R*_Ω_), in series with charge transfer resistance, *R*_ct_, and Warburg W element, in parallel with a constant phase element (CPE).

Electrochemical impedance spectroscopy (EIS) was applied in order to obtain information regarding the interfaces modified by thin films previous described, as well as to evaluate the influence of caffeine on each condition. Spectra were recorded in BRB solution pH 2.0, at open circuit potentials (OCP) and oxidation potentials (*E*_*O*_′) for bare and each modified system, being adjusted by using a modified Randles model (according to Figure 3) composed by the cell resistance (*R*_Ω_), in series with a combination of charge transfer resistance, *R*_ct_, followed by an Warburg element (W), both in parallel with a constant phase element (CPE). The OCP values (in V vs Ag|AgCl) were as follows: bare = 0.153; EL1 = 0.153; EL2 = 0.222; EL3 = 0.133 and EL4 = 0.129. The *E*_*O*_′ values (in V vs Ag|AgCl) were as follows: bare = 1.83; EL1 = 1.53; EL2 = 1.69; EL3 = 1.67; EL4 = 1.72.

**Figure 4:**
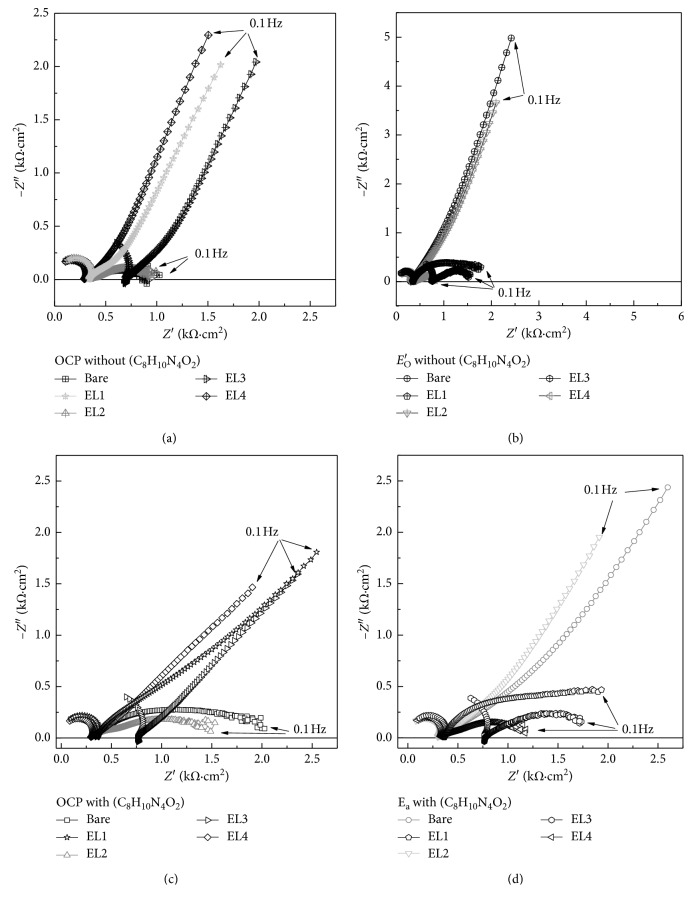
Complex plane spectra recorded for experiments carried out in solution pH 2.0 where (a) is OCP in absence of caffeine, (b) OCP in absence of caffeine for each electrodes recorded at *E*_*O*_′ , (c) OCP in presence of 2.0 mmol·L^−1^ of caffeine for each electrodes, and (d) complex plane spectra recorded for *E*_*O*_′ in presence of 2.0 mmol·L^−1^ of caffeine for each electrode.

Impedance spectra are shown in Figure 4, recorded at composite electrodes: bare and modified EL1, EL2, EL3, and EL4. All spectra present a semicircular profile with notably higher impedance in absence of caffeine. However, these values decrease reaching almost two times lower once the caffeine is introduced at 2.0 mmol·L^−1^.

The region of intermediate frequencies is associated with the transfer of charge at the interface, *R*_ct_. The corresponding relaxation effect is presented in the complex plane with a half-circle presented in all spectra recorded, whose time constant is given by the product *R*_ct_*C*_dl_. Two parameters are obtained from the semicircle analysis, the *R*_ct_, which is the measure of the diameter and the characteristic relaxation frequency, obtained at the maximum value of the semicircle [36].

At low frequencies, it is observed for modified electrodes that the impedance is characterized by diffusion mass transport processes. Two regions can be identified in the complex impedance plane and are characterized by a linear region with phase angle corresponding to the semi-infinite diffusion and represented by Warburg impedance (W). A second linear region still at low frequencies with an angle of phase associated with a purely capacitive response that can be considered electrode reaction, where the slowest step is related to the ionic transport towards the interface. It can be reasonably considered the kinetics of the reaction is limited by diffusion, and this is more expressive when the caffeine is added solution which results in decreases of impedance characterized lowest *R*_ct_ measured [36, 37].

According to the model used, the CPE simulates a nonideal capacitor representing the double layer capacitance, described by CPE = {(*C iω*)^*α*^}^−1^, where *C* is the capacitance and *α* an exponent variable between 1.0 for a smooth/homogeneous surface and 0.5 for a porous/heterogeneous electrodes surfaces [38, 39]. This circuit is appropriate, since the electrode was modified by layers of different films with rough and heterogeneous surface [39, 40].

In this work, the *α* exponent obtained for bare electrodes was *α* = 0.89, and *α* between 0.80 and 0.87 was calculated for the other electrodes. This model predicts that the faradaic current resulting from electronic transfer at the interface is always associated with the capacitive component. The components of the circuit and the different frequency response regions represent the overall electrochemical process. The high frequency region is associated with the resistance of the cell *R*_Ω_ obtaining values between 25 and 35 Ω·cm^2^ for experiments in absence of caffeine and between 25 and 29 Ω·cm^2^ for experiments in the presence of caffeine. These values where compensated. These values were subtracted in the curves in the aim to calculating only the pure values of the respective charge transfer resistances.

Figure 4(a) shows the complex plane diagram for spectra resulted for experiments carried out at respective OCP in absence of caffeine and it is clear to see the electrodes, EL1. EL3 and EL4 have a semicircle followed by a linear profile for low frequencies, indicating process depended from mass transport in the interface of electrodes. Both results present capacitive profile with acclivity of more than π/4°. In the same figure, it is possible to observe two depressed semicircles especially for bare electrode and the second one crossing the *X*-axis, indicating losses on the surface material. However, the profile changes when it is applied at *E*_*O*_′; bare and EL2 electrodes turn to more capacitive profile at low frequencies and the *R*_ct_ decreases for all electrodes, as it shows the Figure 4(b).

Figure 4(c) shows the complex plane plots for experiments carried out with 2.0 mmol·L^−1^ of caffeine where it is clear to see changes on the profiles of spectra with decreases of component at low frequencies for bare EL1 and EL2 electrodes reaching values lower than π/4°.  Figure 4(d) shows the spectra recorded for experiments in presence of caffeine, which are according with the expectation that the *R*_ct_ expressed by the first semicircles equally decreases.  Table 1 presents compilations of charge transfer resistance and double-layer capacitance adjusted by equivalent circuit (Figure 3) for all electrodes.

For samples in the absence of caffeine, polarized at OCP, the charge transfer resistance (Rct) for the bare electrode was 1041 Ω·cm^2^, where the lowest value is obtained for the EL4 with 1/3 of this value at 375 Ω·cm^2^. Since the oxidation potentials of the modifying components of the electrodes are possible surface reactions due to the oxidation of the compounds, it was expected that the *R*_ct_ would suffer a decrease, and in fact, this is observed for the electrodes, bare and EL2, whose *R*_ct_ decreases to values *R*_ct_ lower than 50% of that observed in OCP.

The EL3 maintains its *R*_ct_ basically at the same level; however, EL4, in contrary, increases its charge transfer resistivity, reaching a value higher than observed in the OCP. The double-layer capacitance is maintained in the same order of magnitude, with values *C*_dl_ of 14.9 nF·cm^−2^ for bare electrode and 29.1 nF·cm^−2^ for EL1; no significant changes in their values *C*_dl_ are observed indicating ordering of their surfaces.

In the presence of caffeine in the solution, it is observed for both OCP and oxidation potentials of the modifiers that the resistances measured for EL3 do not undergo significant changes; however, it was observed for the bare and EL2, while for the others, the values remained at the same levels. The double-layer capacitance remains at the same order of values, with emphasis on bare, 26.1 nF·cm^−2^ for OCP and 29.4 nF·cm^−2^. In both potentials, a decrease in the magnitude of the impedances was applied in the presence of caffeine, and a less capacitive behaviour of the electrodes was observed. The electrodes presented stability when submitted to polarization at potentials much higher than the OCP.

The linear characteristic curves observed at lower frequencies are attributed to electron transport limited by diffusion [41, 42]. It can also be observed that the diameter of the first semicircle at the high frequency remains with small alterations by the application of higher potentials; however, the second semicircle is depreciated in the presence of caffeine in solution. This result suggested that the modified electrodes shift their more capacitive pattern to a new profile by decreasing the total impedance values especially in the low saturation loading regime due to the presence of caffeine in solution and consequently, by an increase in the transfer interface.

### 3.6. Electrode Surface Characterization via AFM

The surface profile and roughness of the bare electrode and the 4-benzenesulfonic acid modified electrode were evaluated by AFM, with scans of 10 × 10 *µ*m of the electrodes surface area. All measurements were made in the noncontact mode. In the noncontact mode, the probe does not directly touch the electrode surface, but oscillates above it, measuring the surface topography by the attractive forces between the surface and the probe [27].

**Figure 5:**
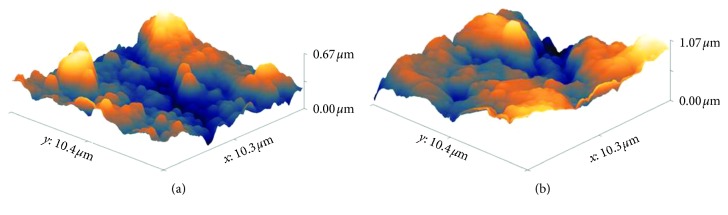
Surface topography via AFM of (a) bare electrode and (b) electrode modified with 4-benzenesulfonic acid.

Root-mean-square (RSM) was used to calculate the average roughness, based on the profile height deviations from the mean line on the AFM image. For both electrodes, three images were recorded on different locations on the surface. For the bare electrode, the RMS was (0.187 ± 0.093), and for the 4-benzenesulfonic acid modified electrode, the RMS was (0.158 ± 0.069). The 3D topography image for each electrode is shown on Figure 5.

From the obtained data, it is possible to infer that the modification leads to a homogeneous surface, with lower roughness when compared to those found for the bare electrode. Such surface change is not itself the unique factor responsible for the improvement noted in respect with sensitivity or selectivity.

### 3.7. Caffeine Determination with 4-Sulfo-benzenediazonium-Modified Electrodes

To evaluate and compare the applicability of the bare and modified electrodes for caffeine determination, an analytical method was developed, for each system, with use of square wave voltammetry (SWV). The parameters pulse amplitude, potential step, and frequency of the square wave signal were optimized to achieve the best overall result, in a solution of 0.04 mol·L^−1^ BRB pH 2 containing 1 mmol·L^−1^ of caffeine (results not shown). The optimized parameters for the bare electrode and the modified electrode were almost the same, with slight difference on frequency. For both systems, 100 mV pulse amplitude and 10 mV potential step were chosen, being the frequencies 8 Hz for the bare electrode and 12 Hz for the modified electrode.

**Figure 6:**
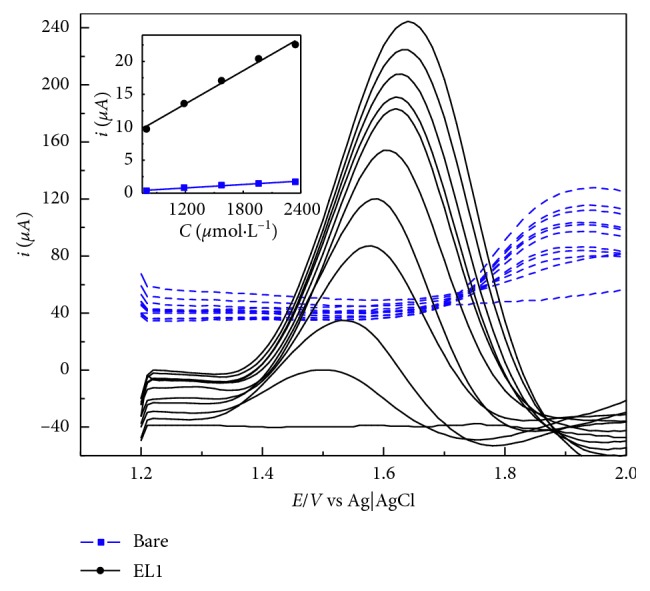
SWV voltammograms showing background measurement followed by 10 additions of 0.1 mL of caffeine standard and 0.1 mol·L^−1^ for bare (dashed lines) and modified electrode (4-benzenesulfonic acid) systems. SWV parameters: 100 mV pulse amplitude, 10 mV potential step, 8 Hz frequency for bare electrode, and 12 Hz frequency for the modified one. Bare electrode curve: *i* = 0.0087 C − 2.45 × 10^−6^; *r*^2^ = 0.9822. EL1 curve: *i* = 0.0837 + 3.51 × 10^−5^; *r*^2^ = 0.9914.

To compare both systems, an analytical curve was made for both systems by spiking a 0.04 mol·L^−1^ BRB pH 2 solution with 10 additions of 0.1 mL of 0.1 mol·L^−1^ caffeine standard.  Figure 6 shows the obtained voltammograms.

The 4-benzenesulfonic acid-modified electrode was more sensitive than the bare electrode, and the oxidation potential of caffeine decreased from (1.87 ± 0.019, *n* = 10) V using the bare electrode to (1.56 ± 0.034, *n* = 10) V onto the modified one. To further optimize the determination method, a smaller concentration curve was made for the modified electrode only, using a caffeine standard with concentration of 5 × 10^−3^ mol·L^−1^. The SWV voltammograms were analogous to the ones shown in Figure 3. In this lower concentration curve, the oxidation potential for caffeine was (1.52 ± 0.013, *n* = 15) V, which represents an average decrease of 0.35 V from the bare electrode oxidation potential.

The analytical parameters for the bare and modified electrodes, including the analytical curves, determination coefficient (*R*^2^), limit of detection (LD), and the linear region (LR), were studied. The LD was calculated based on 10 background measurements. All parameters were calculated for the lowest linear range possible for each system. The obtained parameters for the bare electrode were as follows: curve: *i* = 0.0087 C − 2.45 × 10^−6^; *r*^2^ = 0.9822; LD = 145 *µ*mol·L^−1^; and LR from 800 to 2350 *µ*mol·L^−1^. For the EL1 system, the parameters were as follows: curve: *i* = 0.1163 *C* + 6.34 × 10^−7^; *r*^2^ = 0.9963; LD = 1.3 *µ*mol·L^−1^; and LR from 5 to 150 *µ*mol·L^−1^. The results shown that the modification of the bare electrode with 4-sulfo-benzenediazonium salt increases the sensitivity around 13 times, besides the improvement on the LD.  Table 2 shows a comparison between the proposed method and other methods found in the literature.

### 3.8. Full Validation

The method was validated based on the ANVISA Collegiate Board Resolution (RDC) 166/2017 [28]. The parameters studied were (and the ANVISA RDC 166/2017 limit) as follows: precision (relative standard deviation ≤ 5%), accuracy (recovery 95–105%), linearity (*r* ≥ 0,990), and linear range and detection limit (3.3 times the standard deviation of 10 background measurements divided by the curve slope).

The method linear range, detection limit, and linearity were reported previously. The linear range was 5 to 150 *µ*mol·L^−1^; the detection limit was 1.3 *µ*mol·L^−1^; and the correlation coefficient (*r*) was 0.9981.

To determinate precision and accuracy, twelve determinations were done, on groups of three, each of the 4 samples. The precision was evaluated based on the RSD, and the value obtained was 4.09%. Based on the RDC 166/17, the accuracy was evaluated in comparison with the reference method, and the recovery was 98.37 ± 2.58%.

### 3.9. Caffeine Determination in Tea and Guarana Beverage with Ginseng Aroma Samples

Caffeine was determined in three tea samples, being two black teas and one green tea, and one guarana beverage with ginseng aroma, by the voltammetric method developed and HPLC assays with UV-Vis detector for comparison. All samples were studied in triplicate, and the average results were compared between voltammetric and HPLC via the Student *t*-test, within 95% confidence. The samples were prepared as described in Section 2.5. All voltammetric determinations were made using the 4-benzenesulfonic acid modified electrodes (EL-01).

**Figure 7:**
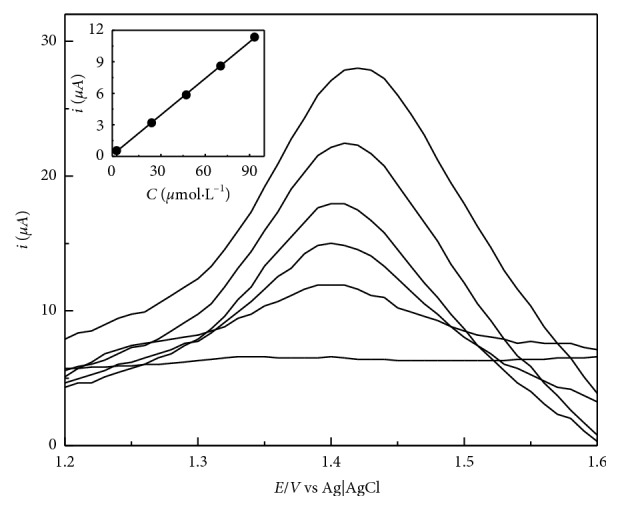
Voltammograms obtained for one of the sample measurements. From bottom to top, it shows a blank measurement (20.0 mL of a 0.04 mol·L^−1^ BRB pH 2 solution), followed by an addition of 0.5 mL of the sample, and four caffeine spikes of 0.1 mL from a standard of 5 × 10^−3^ mol·L^−1^. SWV parameters: frequency of 12 Hz, pulse amplitude of 100 mV, and potential step of 10 mV. Standard addition curve: *i* = 0.1156 + 4.74 × 10^−7^; *r*^2^ = 0.998.

All voltammetric determinations were carried out by standard addition, as aforementioned in the experimental section. For HPLC assays, an analytical curve was built, and all sample concentrations were quantified using this calibration model.  Figure 7 shows an example of the voltammograms seen in a sample determination.  Table 3 comprises all the average concentrations obtained for both voltammetric and HPLC methods. The results were compared via the Student *t*-test and showed no statistical difference.

## 4. Conclusions

Different chemical modifiers were successfully synthesized and characterized for electroanalytical purposes. Modifications were easily carried out by cyclic voltammetry, being the surfaces then assessed by electrochemical impedance spectroscopy, allowing a better understanding of the systems.

The use of the abovementioned chemically electrodes showed to be a simpler method than HPLC; besides this, the proposed procedures also displayed other advantages: the method does not need the filtering step for the sample preparation; there was no need for use of organic solvent, being all experiments done in aqueous solutions only. Additionally, every triplicate determination would take around 10 to 15 minutes to be measured via voltammetry, while in HPLC, every triplicate takes at least 45 minutes.

Modifications led to a relevant shift on peak potentials and to higher sensitivities during caffeine determination in many different samples. The voltammetric method based on such modified electrode was not only cheaper and faster to prepare and measure, but also was more environmental friendly.

According to EIS studies, decreases in magnitude of the impedances in the presence of caffeine depressing the usual capacitive behaviour indicating a linear characteristic curves observed at lower frequencies correlated with electron transport limited by diffusion. Also, shifts are observed in resistances of charge transfer especially in the second semicircle depreciated in the presence of caffeine in solution.

All result suggested that the modified electrodes changes their surface characteristic profile, generally more capacitive, decreasing the total impedance values especially in the low saturation loading regime due to the presence of caffeine in solution and consequently, by an increase in the transfer Interface.

The surface roughness and topography were evaluated with use of AFM images. The images showed a decrease in the surface roughness after modification of the electrodes with diazonium salts. Based on this data, it is possible to infer that the modification made a more homogeneous surface compared with the bare electrode.

## Figures and Tables

**Table 1:** Charge transfer resistance and capacitance obtained by adjusting the spectra using equivalent circuit (Figure 3) for experiments carried out in absence and presence of caffeine at 2.0 mmol·L^−1^ for all electrodes.

	OCP		*E*_*O*_′	
	*R*_ct_ (Ω·cm^2^)	*C*_dl_ (nF·cm^−2^)	*R*_ct_ (Ω·cm^2^)	*C*_dl_ (nF·cm^−2^)
Bare	1041	14.9	443	25.1
	429	26.1	381.0	29.4
EL1	418	29.1	464	26.2
	410	21.3	410	32.3
EL2	940	10.9	505	20.3
	395	28.4	370	30.3
EL3	806	23.1	881	19.3
	830	24.3	829	20.5
EL4	375	27.4	453	26.8
	385	29.1	371	35.6

**Table 2:** Comparison between main reports from the literature and the proposed method, related to carbon-modified electrodes.

Electrode material	Modification	Tech	LD (*µ*mol·L^−1^)	Ref.
Glassy carbon	Poly(4-amino-3-hydroxynaphthalene sulfonic acid)	SWV	0.79	[23]
Glassy carbon	Nafion/ruthenium oxide pyrochlore	SWV	2.20	[43]
Epoxy-graphite composite	4-Benzosulfonic acid	SWV	1.3	This work

**Table 3:** Caffeine determination via voltammetric and HPLC methods in real samples (*n* = 3).

Sample	Voltammetry (mg·L^−1^)	HPLC (mg·L^−1^)
Black tea 1	264.28 ± 15.08	280.01 ± 10.73
Black tea 2	345.31 ± 12.54	340.10 ± 6.15
Green tea	430.21 ± 3.67	433.56 ± 1.32
Guarana drink	65.26 ± 4.03	66.36 ± 1.26

## Data Availability

The data used to support the findings of this study are included within the article.
